# Induced Sputum Substance P in Children with Difficult-to-Treat Bronchial Asthma and Gastroesophageal Reflux: Effect of Esomeprazole Therapy

**DOI:** 10.1155/2011/967460

**Published:** 2011-12-27

**Authors:** Adel Salah Bediwy, Mohamed Gamal A. Elkholy, Mohammed Al-Biltagi, Hesham Galal Amer, Eman Farid

**Affiliations:** ^1^Chest Department, Faculty of Medicine, Tanta University, Tanta 1084, Egypt; ^2^Paediatric Department, Faculty of Medicine, Tanta University, Tanta, Egypt; ^3^Internal Medicine Department, Faculty of Medicine, Minoufiya University, Shebeen El-Koom, Egypt; ^4^Microbiology and Immunology Department, Faculty of Medicine, Benha University, Benha, Egypt

## Abstract

*Objectives*. To assess the induced sputum substance P (ISSP) levels in children having difficult-to-treat asthma (DA) with and without gastroesophageal reflux (GER). We aimed also to evaluate the association of GER with childhood DA, relationship of GER severity with childhood asthma control test (C-ACT), FEV_1_, peak expiratory flow (PEF) variability, and ISSP. Finally, we tried to evaluate esomeprazole treatment effect on C-ACT and FEV_1_ in children with DA. *Methods*. Spirometry, C-ACT, upper gastrointestinal endoscopy, and ISSP measurement were done for children with DA compared to healthy controls. *Results*. ISSP was high in DA with higher levels in the group having associated GER. In the latter group, ISSP and C-ACT improved significantly after esomeprazole treatment while FEV_1_ and PEF variability did not improve. Reflux severity was positively correlated with ISSP and negatively correlated with FEV_1_. *Conclusions*. GER was found in 49% of our patients with childhood DA. Very high ISSP levels in children with DA may be used as a marker for presence of GERD. Esomeprazole therapy improved asthma symptoms but did not improve lung function.

## 1. Introduction

Asthma is a common chronic complex inflammatory airway disorder characterized by variable degrees of recurring symptoms of airflow obstruction and bronchial hyperresponsiveness [1]. Although the majority of asthma patients can obtain the targeted level of control, some patients will not achieve control even with the best therapy [2]. Patients who do not reach an acceptable level of control with the use of reliever medication plus two or more controllers can be considered to have difficult-to-treat asthma [3].

The association between asthma and gastrooesophageal reflux (GER) has been debated for decades when Sir William Osler first observed the association between worsening asthma and distended stomach in 1892 [4]. The prevalence of symptoms of GER among individuals with asthma is substantially higher than in normal population and similarly the prevalence of asthma in individuals with GER is also higher than in controls [5].

Gastroesophageal reflux (GER) may cause chronic respiratory disease by vagal response and tracheal aspiration of gastric contents [6]. Aspiration of gastric contents changes pulmonary resistance and causes reactive airway obstruction [7]. Gastrooesophageal reflux may contribute to airway inflammatory events, possibly by sensory nerve stimulation and the subsequent release of tachykinins into the airway [8].

The tachykinins as substance P (SP) and neurokinin A are the neuropeptides most often associated with axonal reflexes and are potent mediators of cough, bronchospasm, microvascular leakage, and mucus secretion [9]. Asthmatic patients have reduced lower esophageal sphincter pressure and take longer to perform esophageal clearance [10]. In addition, asthma medications such as beta agonists and theophyllin reduce lower esophageal sphincter pressure and increase gastric acid secretion [6, 11].

The prevalence of GERD in children with asthma has been shown to be between 19.3% and 80% in different studies [12]. The diagnosis of GERD is not easy in patients with difficult-to-treat asthma as GER may present solely with respiratory manifestations (occult GER). In children, GER can present with bronchiolitis, pneumonitis, and even failure to thrive. Other common GER respiratory manifestations are chronic coughing, asthma, laryngeal spasm, apnea, stridor, pulmonary dysplasia, and cyanotic crises. Nocturnal wheezing or coughing, with inadequate response to medical treatment for asthma, negative family history of atopy and early onset of bronchial hyperreactivity can distinguish these patients [13]. So, there is a need for a simple test to predict the presence of GERD among children with difficult-to-treat asthma.

We hypothesized that measurement of induced sputum levels of substance P (SP) can be a helpful tool to anticipate presence of GERD in children with difficult-to-treat asthma. So, we aimed to assess the induced sputum substance P (ISSP) levels in children having difficult-to-treat asthma (DA) with and without gastro esophageal reflux (GER). We aimed also to detect the association of GER with the difficult-to-treat asthma in children, as well as to determine the relationship between severity of GER as assessed by upper GIT endoscopy and the childhood asthma control test (C-ACT), forced expiratory volume in the 1st second (FEV_1_), peak expiratory flow (PEF) variability and induced sputum levels of SP. Also this study aimed to determine the effect of antireflux therapy with esomeprazole as proton pump inhibitors (PPI) on the C-ACT, FEV_1_, PEF variability, and induced sputum levels of SP in children having difficult-to-treat asthma with GERD.

## 2. Patients and Methods

**Figure 1:**
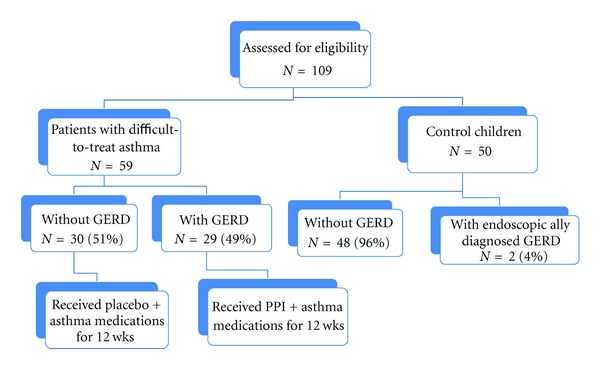
The study design, groups, and subgroups.

Figure 1 showed the study design, where 59 children between 5 and 11 years of age (diagnosed with difficult-to-treat bronchial asthma) and attending the Asthma Clinic of Pediatric and Pulmonology Department at International Hospital of Bahrain, a tertiary care hospital, Kingdom of Bahrain. Patients were identified from the clinic database according to the guidelines of the National Asthma Education and Prevention Program [1] from January 2009 to January 2011. Fifty healthy children of matched age and sex were studied as a control group.

### 2.1. Defining the Cases

Asthma was defined on the basis of symptoms together with documented reversible airflow obstruction (increase FEV_1_ by more than 12% after inhaled short-acting *β*_2_-agonist) and PEF variability ≥20% [14]. Asthmatic children fulfilled the criteria for difficult-to-treat asthma, that is, all had persistent refractory symptoms, were receiving maintenance therapy of inhaled steroids (≥400 *μ*g beclomethasone dipropionate or equivalent per day) and long-acting *β*_2_-agonist and had received at least one course of systemic steroids in the preceding 12 months [15].

Patients with difficult to treat asthma were further subdivided into 2 subgroups according to the presence of signs of GERD on upper GIT endoscopy.

- Children with difficult-to-treat asthma and GERD.
- Children with difficult-to-treat asthma without GERD.

All asthmatic patients were managed according to GINA (Global Initiative for Asthma) guideline 2008 with treatment being stepped up and down as appropriate.

### 2.2. The Following Was Done for All Subjects

Detailed history taking and thorough clinical examination with special stress on GIT symptoms suggestive of reflux including heart burn, acid regurgitation and food regurgitation. In asthmatic patients Childhood Asthma Control Test (C-ACT) was done. It is a seven-item child- and caregiver-completed tool with a scoring range of 0–27; higher scores indicate better control. A score of 19 or less indicates that the asthma may not be well controlled. The C-ACT is intended for use in children up to the age of 12 years [16]. Chest X ray posteroanterior and lateral views were done to exclude other pulmonary diseases (e.g., TB, bronchiectasis, cystic fibrosis, congenital anomalies, etc.). Abdominal ultrasonography was done to exclude organomegaly. Spirometry using calibrated computerized machine (Jaeger MasterScreen-Body/Diffusion, Jaeger, Germany) was done for all cases with special stress on FEV_1_, and peak expiratory flow (PEF) variability. The PEF variability was calculated as the percentile ratio of the difference between maximum and minimum PEF to the mean daily PEF over a period of one week, that is, (maximum PEF − minimum PEF)/(mean of all PEFs over 1 week) × 100 [17]. 

#### 2.2.1. Upper Gastrointestinal Endoscopies Were Done

by using an Olympus GIF-P140 Pediatric Video Gastroscope with 8.5 mm diameter, and 2.2 mm channel. The endoscopic findings of esophagitis were classified according to Los Angeles classification to the following grading: Grade A (score 1): 1 or more mucosal breaks each ≤5 mm in length. Grade B (score 2): at least one mucosal break >5 mm long, but not continuous between the tops of adjacent mucosal folds. Grade C (score 3): at least one mucosal break that is continuous between the tops of adjacent mucosal folds. Grade D (score 4): mucosal break that involve at least three-fourth of the luminal circumferences. Patient took score 0 if there were no features of reflux [16]. Upper gastrointestinal endoscopy was not done in the control group except in 2 cases, because an informed consent could not be obtained from the families to do such invasive technique. The two cases that had been examined with endoscopy had a strong clinical history of GERD.

#### 2.2.2. Induced-Sputum Production

Sputum was collected either spontaneously or induced with hypertonic saline nebulization from all subjects. Prior to sputum induction, children inhaled 200 *μ*g of salbutamol to minimize broncho-constriction during the induction procedure. Sputum was induced by inhalation of 3% hypertonic saline solution for 5 min using an ultrasonic nebulizer, and the subjects were encouraged to cough and expectorate sputum into sterile containers. FEV_1_ was measured after nebulization. Nebulization was stopped if a fall in FEV_1_ of >20% compared to baseline values occurred or if troublesome symptoms appeared [18].

#### 2.2.3. Sputum Substance P (SP) Measurement

SP was measured using a commercially available enzyme linked immunosorbent assay (ELISA; R and D Systems, Oxon, UK). It has no significant cross-reactivity with neurokinin A, neurokinin B, or neuropeptide K. The limit of detection of this assay is 0.06 ng/mL.

#### 2.2.4. The Group of Children Who Had Difficult-to-Treat Asthma with Reflux

Further received medical treatment for reflux in the form of proton pumps inhibitors (PPI) for 12 weeks (esomeprazole capsule 20 mg/day) beside the usual antiasthma medications as mentioned before. *The group of children who had* difficult-to-treat asthma without reflux received placebo identical appearing capsules containing lactose (placebo capsule/day) for 12 weeks beside the usual antiasthma medications as mentioned before.

All children proved to have GERD either asthmatic or the control group were screened for *Helicobacter Pylori* infection and the positive cases received metronidazole and clarithromycin beside the esomeprazole (triple therapy).

So, all asthmatic patients with GERD received PPI and none of them received placebo because it is unethical to give placebo to them instead of proper treatment and also because if we divide them into 2 subgroups, the number of patients in each of them will be low and the power of work would be reduced.

On the other hand, we gave placebo to asthmatic patients without GERD to rule out the placebo effect on improvement in the other group and to exclude the effect of better patient adherence to prescribed medications and better follow up by regular attendance to the clinic.

### 2.3. After 12 Weeks the Following Evaluations Were Repeated

Upper GIT endoscopy; for the group who received antireflux treatment, C-ACT, pulmonary function testing, and substance P measurement in induced sputum for both groups of patients with difficult-to-treat asthma.

Parents of all patients and control subjects signed a written informed consent before enrolment into the study. The local Institutional Research Ethics Committee approved the study protocol.

### 2.4. Statistical Analysis

The power level of the number of cases in the study was more than 80%. Statistical analysis was performed with Statistical Package for Social Sciences (SPSS), version 16.0 (Chicago, IL, USA). Data are presented as mean (±SD) values. Comparison between the studied groups was performed with Students *t*-test, with *P* < 0.05 considered statistically significant. Wilcoxon's signed rank test was used to assess the normality of distributions of the data. The Bonferroni correction/adjustment procedure was done to avoid “significance” due to chance only, in multiple comparison with many parameters. Correlation between variables was evaluated using Pearson's correlation coefficient.

## 3. Results

The study design and the demographic data of patients and the control subjects as well as their clinical data are shown in Figure 1 and Table 1. There was no significant difference in the age and sex between the patients group and the control. However, the body mass index (BMI) was significantly lower in the patient group than the control children. Table 1 also showed that the blood eosinophils, PEF variability, and sputum SP were significantly higher in children with difficult-to-treat asthma than the control children, but FEV_1_ (% of predicted) and C-ACT was significantly lower in children with difficult-to-treat asthma than the control.

Table 2 showed the demographic and clinical data of the children with both difficult-to-treat asthma and GERD (29 children) and those children who have difficult-to-treat asthma with no GERD (30 children). There was no significant difference in age, sex, BMI, age at diagnosis, C-ACT, PEF variability, and FEV_1_ (% of predicted) between the two subgroups. However, the sputum SP was significantly higher in children with difficult-to-treat asthma and GERD than in those who have difficult-to-treat asthma without GERD.

Table 3 showed a significant positive correlation between reflux severity score and induced sputum SP. There were significant negative correlations between FEV_1_ and both reflux severity score and induced sputum SP. C-ACT had significant negative correlations with both reflux severity score and induced sputum SP.

Table 4 showed the effect of 12 weeks treatment with esomeprazole on children with difficult-to-treat asthma and GERD. It showed significant improvement of C-ACT and significant reduction of sputum SP after treatment than before treatment. However, FEV_1_ (% of predicted) and PEF variability showed no significant changes. This table also showed no significant effect of the placebo treatment on children with difficult-to-treat asthma without GERD.

As mentioned in the methodology section, management of asthma was done according to GINA guideline 2008 with treatment stepped up and down as required. Among the 59 children patients with difficult-to-treat asthma, seven patients required stepping up of asthma therapy (three with GERD and four without GERD) while eight patients required stepping down (four with GERD and four without GERD).

**Figure 2:**
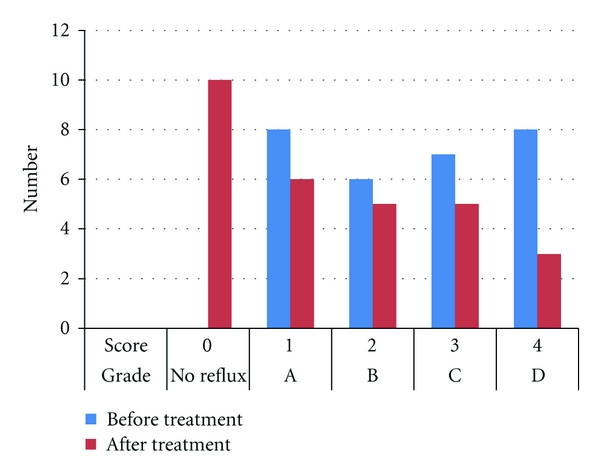
Effect of 12 weeks of treatment with esomeprazole on reflux grade in patient group with difficult-to-treat asthma and GERD.

Figure 2 showed the endoscopic reflux scores in children with difficult-to-treat asthma and GERD. Eight patients (27.59%) had grade A, six patients (20.69%) had grade B, seven patients (24.13%) had grade C, and eight patients (27.59%) had grade D. After 12 weeks of esomeprazole treatment, six patients (20.69%) had grade A, five patients (17.24%) had grade B, five patients (17.24%) had grade C, three patients (10.35%) had grade D, and ten patients (34.48%) showed no endoscopic signs of reflux (Figure 2). Twenty-four children (82.7%) had improvement in reflux score after PPI treatment. Only five children (17.3%) showed no improvement in endoscopic reflux score after treatment, three of them were grade D and remained the same after treatment and two of them were grade C and remained the same after treatment. Two cases among the nonresponders were proved to have *Helicobacter pylori* infection. Also the two control children who had GERD had concomitant infection with *Helicobacter pylori*.

## 4. Discussion

Because of the high incidence of gastro-esophageal reflux (GERD) in patients with asthma [12], the complex relationship between them, and finally the difficulty of diagnosing GERD among asthmatic patients we designed this study. GERD may simply represent just an associated unrelated finding with asthma, it may worsen the severity of asthma, or could be a consequence of asthma itself [19]. According to the best of the authors' knowledge, this is the first work that use induced sputum substance P in assessment of gastro-esophageal reflux (GERD) in children with difficult-to-treat asthma. 

The wide difference in GERD prevalence from one study to another (between 19.3% and 80% in different studies) depends on the method of reporting of GERD (pH monitoring, scintigraphy, endoscopy, and radiologic options) and on the patients' criteria of the studied groups [12]. 

One of the points that we were concerned about in the current study was the association of GERD in a relatively homogenous group of children with difficult-to-treat asthma. Children with difficult-to-treat asthma and GERD made up to 49% of the studied patient group. Kwiecien et al. 2011 found that the intensity of GER was significantly correlated with severity and the difficulty-to-control asthma attacks in asthmatic children [20]. Their finding can explain the high prevalence of GERD in the current study. In the current study, there was a positive correlation between endoscopic reflux severity score (as an indicative of GERD severity) and the asthma severity (as indicated by lower C-ACT, FEV_1_, and the higher PEF variability and sputum SP). With increasing severity of asthma, there is a need to increase the dose and the number of antiasthma medication. In the current study, all the asthmatic children were receiving long-acting Beta-_2_ agonists and high-dose inhaled corticosteroids. Asthma medications such as beta agonists and theophyllin reduce lower esophageal sphincter tone and increase gastric acid secretion [7]. Systemic steroids have been shown to increase GERD in asthma patients [11]. The asthma itself may predispose to the occurrence of GERD by increasing pressure gradient between the thorax and the abdomen with reduction of lower esophageal sphincter pressure and lengthening of the time needed to perform esophageal clearance [21]. On the other hand, the reflux is an important asthma trigger. The potential mechanisms include a vagally mediated esophageal tracheobronchial cough reflex, a local axonal reflex, heightened bronchial reactivity, and micro- or macroaspiration into tracheobronchial tree [10, 22]. 

Being a common finding in children with difficult-to-treat asthma, presence of GERD must be ruled out in such cases. In the current study, there was a significant increase of sputum SP in children with difficult-to-treat asthma and GERD than both control children and children with difficult-to-treat asthma without GERD. In addition, the level of sputum SP significantly decreased in children with difficult-to-treat asthma and GERD after 12 weeks of treatment with the proton pump inhibitor (PPI) esomeprazole. This agreed with the work of Patterson et al. 2007, who found that sputum SP and neurokinin A (NKA) were significantly higher in adult patients with reflux than those without reflux. Also they found significantly higher levels of sputum SP and NKA in adult asthmatic patients with reflux than in adult asthmatic patients without reflux. They suggested that acid in the esophagus caused sensory nerve stimulation and release of tachykinins into the airways [8]. Liu et al. 2005, also found that SP concentration was high in airways of adult GERD patients with cough [23]. Substance P is one of the tachykinins peptides that can cause many pathophysiological features of neurogenic inflammation and is secreted from sensory airway nerves and inflammatory cells after allergens exposure [9]. They are potential mediators of asthma through their potent effects on the bronchomotor tone, airway secretion, bronchial circulation (vasodilatation and microvascular leakage) as well as on inflammatory and immune cells [24]. From the results of the current study as well as the study of Patterson et al. and Liu et al. we can expect presence of associated GERD in children with difficult-to-treat asthma if the sputum SP level is significantly high. However, we did not define a cutoff value of sputum SP with the highest sensitivity to expect presence of GERD in asthmatic children. 

In the current study, childhood-asthma control test (C-ACT) in patients with both difficult asthma and GERD showed significant increase after 12 weeks of treatment with esomeprazole. This agreed with a number of studies done in asthmatics with GERD. Khoshoo and Haydel, 2007, showed a significant improvement in asthma symptoms and decreased requirement for asthma medication in 25 nonatopic asthmatic children treated with acid suppressor treatment [25]. Khoshoo et al. 2009, found that continued treatment with a proton pump inhibitor/prokinetic combination in children with moderate-persistent asthma and concomitant GERD had shown significant clinical improvement in asthma symptoms and no exacerbations for more than 3 months [26]. Yüksel et al. 2006, showed that GERD therapy with famotidine significantly decreased respiratory symptoms in preschool children with asthma [27]. Another study done by Yoshida et al. 2008, showed that the anti-GERD treatment significantly improved bronchial hyperreactivity as indicated by methacholine challenge test in thirty nonatopic children with persistent asthma [28]. Other studies done in adult asthmatics also agreed with the result of the current study. Harding et al. 1996, found that omeprazole improved asthma symptoms in 67% of asthmatics with GERD after 3 months of therapy [29]. Calabrese et al. 2005 found that treatment with pantoprazole for 6 months caused significant improvement of asthma symptoms and FEV_1_ in the adult asthmatics [30].

However, Størdal et al. 2005 found that PPI treatment did not improve asthma symptoms or lung functions in children with asthma and GERD. This dissimilarity from the result of the current study may arise from the difference in their studied group which included children with mild or moderate persistent asthma, and relatively well-controlled asthma on daily inhaled steroids; so that further improvement in asthma outcome may be difficult to be obtained. They also assessed the improvement in asthma symptoms using asthma scoring questionnaire which is different from the C-ACT. They used omeprazole 20 mg daily while in the current study we used esomeprazole 20 mg daily [31]. 

Maev et al. 2003, showed that therapy of bronchial asthma associated with GERD using omeprazole in the dose equal to 40 mg per day or esomeprazole in the dose equal to 40 mg per day contributed to a reliable improvement of both pulmonary and esophageal symptoms. However, application of esomeprazole resulted in a faster reduction of bronchial obstruction and gastroesophageal reflux [32]. 

In the current study, despite the significant improvement of C-ACT after GERD treatment with esomeprazole, there was no significant improvement of FEV_1_ and PEF variability. This finding agreed with a number of studies done in both children and adults with asthma and concomitant GERD. Teichtahl et al. 1996, found no significant differences in FEV_1_, FVC, histamine bronchial responsiveness, and diurnal variation of PEFR between placebo and 4 weeks of 40 mg/day of omeprazole treatments in in adult patients with both asthma and GERD [33]. The same finding was documented by Boeree et al. 1998, who also found no beneficial role for intensive antireflux therapy with high-dose omeprazole (40 mg twice daily for 3 months) to improve pulmonary symptoms and function in patients with asthma and chronic obstructive pulmonary disease, who have severe airway hyperresponsiveness despite maintenance treatment with inhaled corticosteroids [34]. Although Levin et al. 1998, found improvement of peak expiratory flow rate and quality of life in asthmatics with gastroesophageal reflux after daily use of omeprazole 20 mg for 8 wk, the increase in FEV_1_ failed to reach statistical significance [35]. Another cross-sectional study done by Kiljander et al. 1999, showed that 8 weeks of treatment with daily omeprazole 40 mg succeeded to reduce nocturnal asthma symptoms, but failed to improve daytime asthma outcome [36]. Lack of improvement in lung functions in asthmatic patients with reflux after acid suppression may be due to presence of nonacid reflux. Weakly acidic and nonacidic reflux events are known to trigger cough events in humans [37]. In addition, distension of the oesophagus by refluxate induces airway protective reflexes [38].

### 4.1. Limitations of the Study

Esomeprazole was the only antireflux therapy used during the study. It may be inadequate especially for treating cases with high reflux score. This is clearly appearing in lack of improvement of the GERD in 3 cases with reflux score of 4. Lack of prokinetic medications use during the study may be an explanation for the nonsignificant improvement in pulmonary functions as nonacidic refluxate still can trigger the airway reflexes. Another limitation in the current study is lack of determination of a cutoff point for sputum SP that can be more sensitive to detect the presence of GERD in such cases. Further studies are needed to determine the effect of poly antireflux therapy on the pulmonary function as well as to determine the cutoff point for sputum SP level with high sensitivity to detect presence of GERD in asthmatic children. Another limitation of the current study is using endoscopy for diagnosing GERD not pH or impedance studies that may be more sensitive as there will be some false-negative cases with the use of endoscopy. The endoscopy used because it was the available tool in our hospital. Finally, lack of control subjects for presence of GERD may be considered as another limitation. 

## 5. Conclusion

Gastro-esophageal reflux (GER) was found in about 49% of patients with childhood difficult-to-treat asthma. Proper GER therapy can improve asthma symptoms as indicated by childhood asthma control test (C-ACT); however, it has insignificant effects on lung function as indicated by FEV_1_ and peak expiratory flow variability. Very high level of induced sputum substance P (SP) in children with difficult-to-treat asthma may be used as a novel marker for diagnosing GER in such children.

## Figures and Tables

**Table 1:** Demographic data, associated comorbidities, FEV_1_, PEF variability, and induced sputum SP in patients and control group.

	Asthmatic (*n* = 59)	Control (*n* = 50)	*t*	*P*
Age	8.2 ± 1.85	8.0 ± 1.7	0.4	>0.05
BMI	18.4 ± 1.4	20.6 ± 2.3	6.9	<0.001*
M/F	1.1 : 1	1.2 : 1	0.2	>0.05
Age at diagnosis	4.7 ± 1.5	—	—	—
Associated nasal allergy	35 (59%)	4 (8%)		
Atopic dermatitis	24 (41%)	3 (6%)		
Immediate family history of asthma	40 (68%)	2 (4%)		
Smoking parents	13 (22.03%)	12 (24%)		
Long-acting *β*-agonists	59 (100%)			
High-dose inhaled corticosteroids	59 (100%)			
leukotriene modifiers	41 (69.49%)			
Theophylline	35 (59.32%)			
Blood eosinophils (%)	5.9 ± 2.1	2.02 ± 0.9	12.5	<0.001*
FEV_1_ (% of predicted)	58.4 ± 6.98	99.4 ± 5.8	30.6	<0.001*
PEF variability (%)	40.2 ± 4.9	13.2 ± 2.2	34.3	<0.001*
Sputum SP (pg/mL)	1320.9 ± 288.9	584.0 ± 43.9	17.6	<0.001*

BMI: body mass index; EFV_1_: forced expiratory volume in the 1st second; PEF variability: peak expiratory flow variability; sputum SP: sputum levels of substance P.

*P < 0.05 is significant.

**Table 2:** Demographic data, C-ACT, FEV_1_, PEF variability, and induced sputum SP in patient group with difficult-to-treat asthma with and without GERD.

	Difficult-to-treat asthma with GERD *N* = 29	Difficult-to-treat asthma without GERD *N* = 30	*t*	*P*
Age	7.97 ± 1.56	7.96 ± 1.8	0.03	>0.05
Sex M : F	15 : 14	16 : 14		
BMI	20.5 ± 2.5	21.0 ± 2.05	0.88	>0.05
Age at diagnosis	4.7 ± 1.6	4.9 ± 1.4	0.4	>0.05
C-ACT	12.7 ± 3.9	12.8 ± 3.2	0.12	>0.05
FEV_1_ (% of predicted)	57.1 ± 7.9	60.1 ± 4.7	1.8	>0.05
PEF variability (%)	40.7 ± 5.6	39.4 ± 3.6	1.2	>0.05
SP (pg/mL)	1503 ± 84	1004 ± 258	9.2	<0.001*

BMI: body mass index; C-ACT: childhood asthma control test; EFV_1_: forced expiratory volume in 1 second; PEF variability: peak expiratory flow variability; sputum SP: sputum levels of substance P.

*Significant.

**Table 3:** Correlations between studied parameters in asthmatic patients with reflux in children with difficult-to-treat asthma.

Correlation between	*r*	*P*
Reflux severity score and C-ACT	−0.74	<0.001*
Reflux severity score and FEV_1_	−0.64	<0.001*
Reflux severity score and PEFV	0.65	<0.001*
Reflux severity score and induced sputum SP	0.80	<0.001*
Induced sputum SP and ACT	−0.67	<0.001*
Induced sputum SP and FEV_1_	−0.49	<0.001*
Induced sputum SP and PEFV	0.45	<0.001*

C-ACT: childhood asthma control test; EFV_1_: forced expiratory volume in 1 second; PEF variability: peak expiratory flow variability; sputum SP: sputum levels of substance P.

*Significant.

**Table 4:** ACT, FEV_1_, PEF variability, and induced sputum SP in children with difficult-to-treat asthma with GERD treated with esomeprazole and children with difficult-to-treat asthma without GERD treated with placebo before and after treatment.

	Before treatment	After treatment	*t*	*P*
Children with difficult-to-treat asthma and GERD (*n* 29)				

C-ACT	12.7 ± 3.99	15.03 ± 4.4	11.6	<0.001*
FEV_1_ (% of predicted)	57.1 ± 7.9	57.6 ± 7.6	1.2	>0.05
PEF variability (%)	40.7 ± 5.5	40.3 ± 5.2	1.6	>0.05
SP (pg/mL)	1502 ± 83.6	1198 ± 223.5	9.4	<0.001*

Children with difficult-to-treat asthma without GERD (*n* 30)				

C-ACT	12.9 ± 3.3	12.7 ± 2.9	1.97	>0.05
FEV_1_ (% of predicted)	60.2 ± 4.6	60.4 ± 4.5	0.59	>0.05
PEF variability (%)	39.3 ± 3.6	38.9 ± 3.2	1.6	>0.05
SP (pg/mL)	1.68.6 ± 65.3	1067.6 ± 67.1	0.25	>0.05

BMI: body mass index; C-ACT: childhood asthma control test; EFV_1_: forced expiratory volume in 1 second; PEF variability: peak expiratory flow variability; sputum SP: sputum levels of substance P.

*Significant.
